# Differences in receipt of opioid agonist treatment and time to enter treatment for opioid use disorder among specialty addiction programs in the United States, 2014-17

**DOI:** 10.1371/journal.pone.0226349

**Published:** 2019-12-12

**Authors:** Justin C. Yang, Andres Roman-Urrestarazu, Carol Brayne

**Affiliations:** 1 Department of Public Health & Primary Care, Institute of Public Health, University of Cambridge, Cambridge, England, United Kingdom; 2 Department and Epidemiology and Applied Clinical Research, Division of Psychiatry, Faculty of Brain Sciences, University College London, London, England, United Kingdom; 3 Department of International Health, Care and Public Health Research Institute, Faculty of Health, Medicine and Life Sciences, Maastricht University, Maastricht, The Netherlands; Newcastle University, UNITED KINGDOM

## Abstract

**Background:**

Access to adequate treatment for opioid use disorder (OUD) has been a high priority among American policymakers. Elucidation of the sociodemographic and institutional differences associated with the use, or lack thereof, of opioid agonist therapy (OAT) provides greater clarity on who receives OAT. Timely access to care is a further consideration and bears scrutiny as well.

**Methods:**

We draw upon data from the Treatment Episode Data Set—Admissions (TEDS-A) to analyse the relationship between sociodemographic and institutional characteristics and the receipt of opioid agonist treatments and time waiting to enter treatment.

**Results:**

Estimates from logistic regression models highlight certain groups which show lower odds of receipt of OAT, including those in precarious housing arrangements, those unemployed or not otherwise in the labor force, and those referred by drug abuse care providers, educational institutions, employers, and the criminal justice system. Groups which showed higher odds of waiting over a week to enter treatment included those who were separated, divorced, or widowed, those working part-time, and those referred by drug abuse care providers, employers, and the criminal justice system.

**Conclusion:**

Given the efficacy of OAT and the adverse outcomes associated with long waiting times, coordinated effort is needed to understand why these differences persist and how they may be addressed through appropriate policy responses.

## Introduction

In 2010 the global burden of disease attributable to opioid dependence was 9.2 million disability-adjusted life years (DALYs) with 15.5 million individuals suffering from opioid dependence and a significantly high burden of premature mortality affecting North America and Eastern Europe [1]. In 2015, over 33,000 deaths from overdoses were recorded in the United States, nearly equal to the number of deaths from traffic accidents for the same period, with deaths from heroin alone exceeding those from homicides involving firearms [2]. The opioid epidemic in the United States has been one of the most pressing public health challenges identified by the United States Centers for Disease Control and Prevention (CDC) [2], involving both heroin use, proved to be exacerbated by socioeconomic vulnerability [3], as well as ease of accessibility and over prescription of synthetic opioids such as oxycodone and fentanyl, respectively, which appear to fuel the increasing toxicity and mortality of these substances [4]. The effects of this increasing prevalence has been an upsurge in opioid-related overdose deaths that have tripled between 1999 and 2014, with 60.9% of drug-related deaths involving an opioid [5]. Moreover, use disorders involving prescription and synthetic opioids has steadily increased; from 1997 to 2011, the number of individuals seeking treatment for opioid addiction increased by 900% [2]. Despite the urgent need for additional capacity and health system responsiveness for opioid use disorder (OUD) treatment, including the need for qualified care providers and available space in substance abuse treatment facilities, individuals with OUDs continue to face barriers to evidence-based treatment such as psychotherapy and opioid agonist treatments (OATs) which are established best practice [6, 7]. One national study in 2013 found, for instance, that lifetime cumulative probability of treatment-seeking among individuals with opioid addiction was only 42% with a median delay of 3.83 years from onset of disorder to first treatment [8]. A more recent study has also highlighted racial and ethnic differences in OAT for OUD which signal a greater need for focus to understand and overcome potential barriers to treatment to promote health equity [9]. These findings, in conjunction with research that show that opiate-dependent patients waiting for treatment are at heightened risk for mortality [10], indicate a need for greater scrutiny of barriers to treatment. In addition to barriers to treatment, the type and mode of treatment received by individuals with OUD has also been at the centre of the access barriers debate [11, 12]. OATs, such as methadone [13], are cost-effective, evidence-based treatments for OUD, especially compared against abstinence-based treatments [13, 14]. Nevertheless, OATs have historically been subject to heightened scrutiny in the United States; for example, the use of methadone is strictly regulated by the Drug Addiction Treatment Act (DATA) of 2000 and limited only to certified Opioid Treatment Programs (OTPs) [15]. Given the stringent regulatory oversight of OATs for the treatment of OUD amid the opioid crisis, the accessibility of OATs and the capacity to treat OUD has come under heightened scrutiny [16] with some calling for increased access to buprenorphine in the outpatient setting [17]. Moreover, given the urgency of non-medical use of prescription opioids (NMUPO), some attention has also been devoted to the timely receipt of care for OUD [18].

Our aim was to examine and identify patients with OUD in specialty addiction programs at risk of not receiving OAT as well as delayed entry to treatment based on: sociodemographic, and institutional characteristics. In this study, we draw upon the Treatment Episode Dataset (TEDS), an administrative dataset of annual admissions to substance abuse treatment facilities to analyse the differential use of OAT among admitted patients by patient characteristics as well as factors underlying time to enter treatment.

## Material and methods

### Data source

We used data from the Treatment Episode Data Set—Admissions (TEDS-A), a national administrative, fully anonymized dataset coordinated and maintained by the Center for Behavioral Health Statistics and Quality at the Substance Abuse and Mental Health Service Administration (SAMHSA), for admissions from 2014–17 [19]. TEDS-A captures information at intake on all publicly-funded admissions to public and private substance abuse treatment facilities in all 50 States, the District of Columbia, and Puerto Rico, as well as some privately-funded admissions to facilities which receive public funding, depending on whether State regulations require this information or not [19]. The unit of analysis in TEDS-A is admission, not an individual; consequently, an individual may be represented as multiple admissions in TEDS-A [19]. Nevertheless, the TEDS-A data file excludes admissions known to be transfers from one level of care to another within a single treatment episode for the same provider [19]. Collected information includes: sociodemographic characteristics of admitted patients, such as sex, age, and primary source of income, and their substance use behaviours, such as types of substances used, institutional information pertaining to the admission, and indicators of behavioural health of admitted patients [19].

Our analyses included all first-time admissions for opioid treatment where at least one of: heroin, non-prescription methadone, or other opiates was reported as the primary, secondary, or tertiary substance of abuse at time of admission (where TEDS-A only captures up to three substances of abuse at time of admission). Given our interest in the long-term treatment of OUDs with OATs vis-à-vis acute detoxification treatments, we excluded patients who were admitted only for detoxification treatment. As our outcome variables of interest were whether or not an admitted patient received opioid agonist therapy and time waiting to enter treatment, we excluded states which reported no patients receiving opioid agonist therapy (Georgia, Kansas, Montana, North Dakota, Oklahoma, Virginia, and West Virginia) or states missing data regarding time waiting to enter treatment (Connecticut, Georgia, Kentucky, Minnesota, New York, North Carolina, Oklahoma, Oregon, Rhode Island, Vermont, Virginia, Washington, and West Virginia) for each analysis, respectively, as these were likely to represent reporting errors or non-response for optional modules of TEDS-A. On This approach has been adopted elsewhere [20].

### Study variables

Our primary outcome variables were whether an admitted patient received OAT, coded as a dichotomous variable by SAMHSA; and days waiting to enter treatment, coded as an ordinal categorical variable by SAMHSA (i.e. no wait, within one week, within two weeks, within one month, and more than one month). For our analysis of time waiting to enter treatment, we further dichotomized time waiting to enter treatment as either: within one week or greater than one week. This interval was selected given the clinical importance of timely OAT initiation for patients experiencing physiological dependence arising from OUD [21].

Independent variables were categorized as sociodemographic or institutional. Demographic independent variables included age, sex, ethnicity, marital status, living arrangement, and veteran status. Socioeconomic variables included years of education, employment status, primary source of income, and health insurer. Institutional characteristics included service setting at time of admission and primary source of referral. These independent variables have been well-characterized in TEDS-A and used extensively in other comparable analyses [22–27].

In addition to variables already provided in TEDS-A, namely sociodemographic characteristics of admitted patients and institutional characteristics pertaining to admission, we coded for a dichotomous variable to indicate the reported use of alcohol or benzodiazepines on admission, both of which contraindicate the use of OAT for OUD [28] and could confound our analysis of receipt of OAT.

### Statistical analysis

All statistical analyses were performed in Stata 14 [29]. For our analyses of OAT, a dichotomous outcome variable indicating whether an admitted patient received OAT, we conducted multiple maximum-likelihood logit regressions to simultaneously model how sociodemographic characteristics of admitted patients and institutional characteristics pertaining to admission were related to OAT. For our analyses of days waiting to enter treatment, a categorical outcome variable, we conducted multiple maximum-likelihood logistic regressions to simultaneously model how our predictor variables were related to a wait time of over one week to enter treatment.

## Results

Descriptive sample characteristics are presented in Tables 1 and 2. Of the 6,559,735 admissions included in the 2014–17 TEDS-A dataset, 479,322 first-time admissions for OUD treatment were included in our analysis. We note that just over one-third of admitted patients in our sample received OAT and nearly three-quarters of admitted patients were treated with no reported wait time.

**Table 1:** Characteristics of admitted patients either receiving or not receiving opioid agonist therapy for opioid use disorder, 2014–17.

Characteristic	Medication-Assisted Opioid Therapy				
	No		Yes		Total
	No.	%	No.	%	
Age					
18–20	15,850	82.1	3,446	17.9	19,296
21–24	49,224	75	16,399	25	65,623
25–29	76,169	69.2	33,939	30.8	110,108
30–34	56,587	65.4	29,960	34.6	86,547
35–39	35,327	62.5	21,199	37.5	56,526
40–44	20,810	58.1	14,998	41.9	35,808
45–49	16,546	51	15,915	49	32,461
50–54	12,848	45.9	15,165	54.1	28,013
55+	13,449	41.6	18,881	58.4	32,330
Total	296,810	63.6	169,902	36.4	466,712
Sex					
Male	171,350	64.1	95,763	35.9	267,113
Female	125,407	62.9	74,121	37.1	199,528
Total	296,757	63.6	169,884	36.4	466,641
Ethnicity					
White	232,348	67.1	113,809	32.9	346,157
Black or African American	31,093	51.3	29,525	48.7	60,618
Asian or Pacific Islander	2,545	59.1	1,761	40.9	4,306
Native American	5,408	72.7	2,032	27.3	7,440
Other	19,613	57.1	14,753	42.9	34,366
Total	291,007	64.3	161,880	35.7	452,887
Marital Status					
Never Married	161,768	66.2	82,587	33.8	244,355
Married	35,931	64.1	20,163	35.9	56,094
Separated	14,626	72.5	5,556	27.5	20,182
Divorced or Widowed	29,048	68.3	13,510	31.7	42,558
Total	241,373	66.5	121,816	33.5	363,189
Living Arrangement					
Independent	202,019	60.7	130,699	39.3	332,718
Dependent	55,161	73.7	19,643	26.3	74,804
Homeless	29,199	74.6	9,961	25.4	39,160
Total	286,379	64.1	160,303	35.9	446,682
Veteran Status					
No	264,993	63.5	152,523	36.5	417,516
Yes	5,934	63.4	3,430	36.6	9,364
Total	270,927	63.5	155,953	36.5	426,880
Education					
<8 Years	13,790	64.6	7,567	35.4	21,357
9–11 Years	60,295	63.9	34,096	36.1	94,391
12 Years	140,093	64	78,825	36	218,918
13–15 Years	57,293	71.6	22,716	28.4	80,009
16+ Years	15,655	62.8	9,285	37.2	24,940
Total	287,126	65.3	152,489	34.7	439,615
Employment Status					
Full-Time	43,621	61.6	27,175	38.4	70,796
Part-Time	21,843	62.1	13,352	37.9	35,195
Unemployed	132,192	69.8	57,303	30.2	189,495
Not in Labor Force	94,569	61	60,381	39	154,950
Total	292,225	64.9	158,211	35.1	450,436
Source Of Income/Support					
Wages/Salary	46,554	62.1	28,460	37.9	75,014
Public Assistance	12,312	45.9	14,490	54.1	26,802
Retirement/Pension or Disability	9,815	46.3	11,387	53.7	21,202
Other	27,174	60.9	17,427	39.1	44,601
None	68,862	79.9	17,310	20.1	86,172
Total	164,717	64.9	89,074	35.1	253,791
Health Insurance					
Private	11,591	74.8	3,896	25.2	15,487
Medicaid	53,938	45.6	64,462	54.4	118,400
Medicare or Other	12,074	68.4	5,588	31.6	17,662
Uninsured	53,543	82.9	11,060	17.1	64,603
Total	131,146	60.7	85,006	39.3	216,152
Service Setting At Admission					
Hospital	1,735	93.1	129	6.9	1,864
Short-Term	51,556	92.6	4,105	7.4	55,661
Long-Term	30,844	93	2,329	7	33,173
Ambulatory, Intensive Outpatient	54,297	88	7,387	12	61,684
Ambulatory, Non-Intensive Outpatient	158,361	50.4	155,865	49.6	314,226
Total	296,793	63.6	169,815	36.4	466,608
Principal Source Of Referral					
Individual (including Self-Referral)	110,118	45.8	130,248	54.2	240,366
Alcohol/Drug Abuse Care Provider	22,933	72.4	8,750	27.6	31,683
Other Health Care Provider	24,310	76.4	7,513	23.6	31,823
Educational Institution	209	86.7	32	13.3	241
Employer	1,167	92.2	99	7.8	1,266
Other Community Referral	34,836	68.7	15,864	31.3	50,700
Court/Criminal Justice Referral/DUI	96,218	93.8	6,356	6.2	102,574
Total	289,791	63.2	168,862	36.8	458,653
Alcohol or Benzodiazepines Reported at Admission					
No	214,354	58.4	152,996	41.6	367,350
Yes	82,456	83	16,906	17	99,362
Total	296,810	63.6	169,902	36.4	466,712

**Table 2:** Characteristics of admitted patients by days waiting to enter treatment for opioid use disorder, 2014–17.

Characteristic	Days Waiting to Enter Treatment										
	No wait		Within one week		Within two weeks		Within one month		More than one month		Total
	No.	%	No.	%	No.	%	No.	%	No.	%	
Age											
18–20	8,236	68.2	2,551	21.1	627	5.2	482	4	172	1.4	12,068
21–24	29,803	70.7	8,067	19.1	2,119	5	1,631	3.9	516	1.2	42,136
25–29	53,751	72.9	12,964	17.6	3,422	4.6	2,589	3.5	1,009	1.4	73,735
30–34	42,989	73.7	10,138	17.4	2,478	4.2	1,966	3.4	736	1.3	58,307
35–39	28,480	74.5	6,355	16.6	1,655	4.3	1,247	3.3	510	1.3	38,247
40–44	18,507	75.7	4,103	16.8	893	3.7	681	2.8	262	1.1	24,446
45–49	18,219	78.1	3,509	15	815	3.5	593	2.5	196	0.8	23,332
50–54	16,535	79.8	3,000	14.5	586	2.8	461	2.2	150	0.7	20,732
55+	20,012	81.9	3,097	12.7	637	2.6	495	2	193	0.8	24,434
Total	236,532	74.5	53,784	16.9	13,232	4.2	10,145	3.2	3,744	1.2	317,437
Sex											
Male	134,703	74.4	30,936	17.1	7,460	4.1	5,838	3.2	2,179	1.2	181,116
Female	101,799	74.7	22,840	16.8	5,767	4.2	4,304	3.2	1,564	1.1	136,274
Total	236,502	74.5	53,776	16.9	13,227	4.2	10,142	3.2	3,743	1.2	317,390
Race											
White	166,028	72.6	41,247	18	10,417	4.6	8,071	3.5	2,978	1.3	228,741
Black or African American	38,506	79.7	7,103	14.7	1,461	3	986	2	265	0.5	48,321
Asian or Pacific Islander	2,445	73.9	603	18.2	107	3.2	114	3.4	38	1.1	3,307
Native American	2,508	74.6	507	15.1	148	4.4	130	3.9	70	2.1	3,363
Other	16,070	75.7	3,225	15.2	902	4.2	697	3.3	333	1.6	21,227
Total	225,557	74	52,685	17.3	13,035	4.3	9,998	3.3	3,684	1.2	304,959
Marital Status											
Never Married	115,434	71.2	32,037	19.8	7,498	4.6	5,479	3.4	1,729	1.1	162,177
Married	22,734	70.5	6,420	19.9	1,563	4.8	1,131	3.5	379	1.2	32,227
Separated	8,620	70	2,361	19.2	658	5.3	504	4.1	169	1.4	12,312
Divorced or Widowed	18,364	69.3	5,399	20.4	1,383	5.2	1,002	3.8	338	1.3	26,486
Total	165,152	70.8	46,217	19.8	11,102	4.8	8,116	3.5	2,615	1.1	233,202
Living Arrangement											
Independent	158,464	73.8	38,170	17.8	9,192	4.3	6,645	3.1	2,336	1.1	214,807
Dependent	44,128	74.8	9,312	15.8	2,445	4.1	2,193	3.7	890	1.5	58,968
Homeless	20,257	71.5	5,114	18.1	1,393	4.9	1,121	4	446	1.6	28,331
Total	222,849	73.8	52,596	17.4	13,030	4.3	9,959	3.3	3,672	1.2	302,106
Veteran Status											
No	213,260	73.8	50,307	17.4	12,538	4.3	9,446	3.3	3,421	1.2	288,972
Yes	4,869	71.6	1,317	19.4	278	4.1	253	3.7	88	1.3	6,805
Total	218,129	73.7	51,624	17.5	12,816	4.3	9,699	3.3	3,509	1.2	295,777
Education											
<8 Years	9,325	73.8	2,256	17.9	496	3.9	409	3.2	146	1.2	12,632
9–11 Years	48,243	74.2	10,996	16.9	2,730	4.2	2,249	3.5	805	1.2	65,023
12 Years	112,701	73.5	27,332	17.8	6,603	4.3	4,925	3.2	1,829	1.2	153,390
13–15 Years	32,722	69.3	9,233	19.6	2,546	5.4	1,942	4.1	775	1.6	47,218
16+ Years	12,214	76.3	2,536	15.8	660	4.1	459	2.9	140	0.9	16,009
Total	215,205	73.1	52,353	17.8	13,035	4.4	9,984	3.4	3,695	1.3	294,272
Employment Status											
Full-Time	31,458	72.4	8,137	18.7	1,964	4.5	1,375	3.2	518	1.2	43,452
Part-Time	16,544	72.3	4,074	17.8	1,174	5.1	788	3.4	297	1.3	22,877
Unemployed	89,931	72	22,929	18.4	5,956	4.8	4,471	3.6	1,588	1.3	124,875
Not in Labor Force	85,056	76.2	17,899	16	4,017	3.6	3,378	3	1,272	1.1	111,622
Total	222,989	73.6	53,039	17.5	13,111	4.3	10,012	3.3	3,675	1.2	302,826
Source Of Income/Support											
Wages/Salary	38,861	71.4	10,421	19.1	2,612	4.8	1,894	3.5	647	1.2	54,435
Public Assistance	15,807	77	3,352	16.3	666	3.2	529	2.6	167	0.8	20,521
Retirement/Pension or Disability	13,882	76.7	2,996	16.6	577	3.2	475	2.6	167	0.9	18,097
Other	17,382	74.1	4,498	19.2	820	3.5	582	2.5	164	0.7	23,446
None	42,976	66.3	14,550	22.5	3,320	5.1	2,930	4.5	1,013	1.6	64,789
Total	128,908	71.1	35,817	19.8	7,995	4.4	6,410	3.5	2,158	1.2	181,288
Health Insurance											
Private	8,586	61.1	4,105	29.2	735	5.2	485	3.5	140	1	14,051
Medicaid	85,714	80.1	15,646	14.6	2,804	2.6	2,234	2.1	632	0.6	107,030
Medicare or Other	10,251	73.8	2,445	17.6	552	4	466	3.4	167	1.2	13,881
Uninsured	40,396	69.2	12,134	20.8	2,861	4.9	2,313	4	708	1.2	58,412
Total	144,947	75	34,330	17.8	6,952	3.6	5,498	2.8	1,647	0.9	193,374
Service Setting At Admission											
Hospital	85	26.6	122	38.2	42	13.2	50	15.7	20	6.3	319
Short-Term	22,173	64.7	7,710	22.5	2,234	6.5	1,717	5	462	1.3	34,296
Long-Term	14,419	59	5,772	23.6	1,634	6.7	1,760	7.2	856	3.5	24,441
Ambulatory, Intensive Outpatient	33,292	68.2	11,170	22.9	2,307	4.7	1,583	3.2	433	0.9	48,785
Ambulatory, Non-Intensive Outpatient	166,537	79.5	29,007	13.8	7,014	3.3	5,035	2.4	1,973	0.9	209,566
Total	236,506	74.5	53,781	16.9	13,231	4.2	10,145	3.2	3,744	1.2	317,407
Principal Source Of Referral											
Individual (including Self-Referral)	132,257	78.3	25,967	15.4	5,390	3.2	3,830	2.3	1,387	0.8	168,831
Alcohol/Drug Abuse Care Provider	14,981	65.5	5,328	23.3	1,411	6.2	977	4.3	179	0.8	22,876
Other Health Care Provider	11,028	70	3,248	20.6	729	4.6	532	3.4	209	1.3	15,746
Educational Institution	87	68.5	26	20.5	7	5.5	4	3.1	3	2.4	127
Employer	266	65.8	94	23.3	27	6.7	17	4.2	0	0	404
Other Community Referral	26,036	75.1	5,837	16.8	1,464	4.2	1,046	3	301	0.9	34,684
Court/Criminal Justice Referral/DUI	47,123	68.3	12,744	18.5	4,001	5.8	3,570	5.2	1,541	2.2	68,979
Total	231,778	74.4	53,244	17.1	13,029	4.2	9,976	3.2	3,620	1.2	311,647
Alcohol or Benzodiazepines Reported at Admission											
Substance Not Reported	199,639	76.2	41,930	16	9,856	3.8	7,641	2.9	2,861	1.1	261,927
Substance Reported	36,893	66.5	11,854	21.4	3,376	6.1	2,504	4.5	883	1.6	55,510
Total	236,532	74.5	53,784	16.9	13,232	4.2	10,145	3.2	3,744	1.2	317,437

### Use of opioid agonist therapy

The unadjusted and adjusted results of logistic regression of patient and institutional characteristics on receipt of OAT are shown in Table 3.

**Table 3:** Logistic regression estimates for receipt of opioid agonist therapy for opioid use disorder among admitted patients, 2014–17.

Characteristic	Unadjusted		Adjusted*	
	OR	95% CI	AOR	95% CI
Age				
18–20	1		1	
21–24	1.532	(1.471–1.596)	1.527	(1.377–1.693)
25–29	2.049	(1.971–2.131)	1.780	(1.612–1.966)
30–34	2.435	(2.341–2.533)	2.083	(1.884–2.304)
35–39	2.760	(2.650–2.874)	2.365	(2.130–2.625)
40–44	3.315	(3.177–3.459)	2.706	(2.426–3.018)
45–49	4.424	(4.239–4.618)	3.023	(2.706–3.378)
50–54	5.429	(5.197–5.672)	3.024	(2.698–3.390)
55+	6.457	(6.186–6.741)	3.444	(3.068–3.865)
Female	1.058	(1.045–1.070)	1.113	(1.076–1.151)
Ethnicity				
White	1		1	
Black or African American	1.939	(1.905–1.973)	0.987	(0.941–1.035)
Asian or Pacific Islander	1.413	(1.329–1.502)	0.882	(0.733–1.060)
Native American	0.767	(0.729–0.808)	1.355	(1.122–1.637)
Other	1.536	(1.501–1.571)	0.822	(0.734–0.920)
Marital Status				
Never Married	1		1	
Married	1.099	(1.078–1.120)	0.938	(0.896–0.982)
Separated	0.744	(0.721–0.768)	0.835	(0.778–0.897)
Divorced or Widowed	0.911	(0.891–0.931)	0.813	(0.772–0.857)
Living Arrangement				
Independent	1		1	
Dependent	0.550	(0.541–0.560)	0.706	(0.670–0.745)
Homeless	0.527	(0.515–0.540)	0.731	(0.681–0.784)
Veteran Status	1.004	(0.962–1.048)	1.100	(0.988–1.224)
Years of Education				
<8 Years	1		1	
9–11 Years	1.031	(0.999–1.063)	1.185	(1.099–1.278)
12 Years	1.025	(0.996–1.056)	1.061	(0.989–1.138)
13–15 Years	0.723	(0.700–0.746)	0.956	(0.882–1.035)
16+ Years	1.081	(1.041–1.123)	0.905	(0.826–0.992)
Employment Status				
Full-Time	1		1	
Part-Time	0.981	(0.956–1.007)	0.974	(0.915–1.036)
Unemployed	0.696	(0.683–0.708)	0.868	(0.812–0.928)
Not in Labor Force	1.025	(1.006–1.044)	0.770	(0.717–0.827)
Primary Source of Income				
Wages/Salary	1		1	
Public Assistance	1.925	(1.872–1.980)	1.265	(1.175–1.361)
Retirement/Pension or Disability	1.898	(1.840–1.957)	1.241	(1.147–1.343)
Other	1.049	(1.024–1.075)	0.989	(0.923–1.059)
None	0.411	(0.402–0.420)	0.991	(0.928–1.059)
Health Insurer				
Private	1		1	
Medicaid	3.556	(3.423–3.694)	1.676	(1.579–1.779)
Medicare or Other	1.377	(1.312–1.445)	1.348	(1.242–1.464)
Uninsured	0.615	(0.589–0.641)	1.049	(0.986–1.116)
Service Setting at Time of Admission				
Hospital	1		1	
Short-Term	1.071	(0.893–1.284)	4.455	(1.076–18.45)
Long-Term	1.016	(0.845–1.220)	3.986	(0.960–16.56)
Ambulatory, Intensive Outpatient	1.830	(1.528–2.192)	5.420	(1.310–22.42)
Ambulatory, Non-Intensive Outpatient	13.24	(11.07–15.83)	37.69	(9.116–155.9)
Primary Source of Referral				
Individual (including Self-Referral)	1		1	
Alcohol/Drug Abuse Care Provider	0.323	(0.314–0.331)	0.437	(0.410–0.467)
Other Health Care Provider	0.261	(0.254–0.268)	0.512	(0.482–0.543)
Educational Institution	0.129	(0.0892–0.188)	0.114	(0.0438–0.294)
Employer	0.0717	(0.0584–0.0881)	0.101	(0.0636–0.159)
Other Community Referral	0.385	(0.377–0.393)	0.253	(0.241–0.266)
Court/Criminal Justice Referral/DUI	0.0558	(0.0544–0.0574)	0.0686	(0.0651–0.0724)
Alcohol or Benzodiazepines Reported at Admission	0.287	(0.282–0.292)	0.464	(0.445–0.484)

*Adjusted for year, state, age, sex, ethnicity, marital status, living arrangement, veteran status, years of education, employment status, primary source of income, health insurer, census division, service setting at time of admission, primary source of referral, and alcohol/benzodiazepine report at admission.

#### Sociodemographic characteristics

We found that the odds of receipt of OAT were higher in all age groups relative to the reference group (aged 18–20) with the highest odds of receipt of OAT reported by those in age groups 45–49, 50–54, and 55+. Women showed very slightly higher odds of receipt of OAT compared to men. Native Americans showed higher odds of receipt of OAT compared to White Americans while those reporting Other as ethnicity showed lower odds. Compared to those reporting never having married, all other groups showed lower odds of receipt of OAT. Those reporting a dependent or homeless living situation showed lower odds of receipt of OAT compared to those who reported an independent living situation. There was no statistically significant difference in the odds of receipt of OAT between veterans and non-veterans. Compared to those working full-time, those who were unemployed or otherwise not in the labor force exhibited lower odds of receipt of OAT. Those insured by either Medicaid or Medicare showed higher odds of receipt of OAT.

#### Institutional characteristics

Those admitted to short-term care facilities or an ambulatory care setting showed statistically significantly higher odds of receipt of OAT compared to those admitted to the hospital setting. All primary sources of referral showed lower odds of receipt of OAT compared with the reference group of individually referred (including self-referred) patients.

### Days waiting to enter treatment

The unadjusted and adjusted results of logistic regression of patient and institutional characteristics on time waiting to enter treatment (i.e. one week or less compared to more than one week) are shown in Table 4.

**Table 4:** Logistic regression estimates for over one week spent waiting to enter treatment for opioid use disorder among admitted patients, 2014–17.

Characteristic	Unadjusted		Adjusted*	
	OR	95% CI	AOR	95% CI
Age				
18–20	1		1	
21–24	0.949	(0.888–1.013)	1.047	(0.941–1.165)
25–29	0.886	(0.832–0.944)	1.119	(1.009–1.241)
30–34	0.821	(0.770–0.876)	1.077	(0.968–1.198)
35–39	0.825	(0.771–0.883)	1.080	(0.965–1.210)
40–44	0.684	(0.634–0.737)	0.966	(0.854–1.093)
45–49	0.622	(0.576–0.671)	1.069	(0.942–1.213)
50–54	0.516	(0.475–0.560)	0.954	(0.834–1.092)
55+	0.483	(0.446–0.523)	0.889	(0.773–1.022)
Female	0.999	(0.974–1.024)	1.010	(0.968–1.055)
Ethnicity				
White	1		1	
Black or African American	0.574	(0.551–0.598)	0.886	(0.833–0.943)
Asian or Pacific Islander	0.821	(0.722–0.932)	0.924	(0.753–1.134)
Native American	1.115	(0.997–1.246)	0.977	(0.807–1.183)
Other	0.967	(0.921–1.015)	0.980	(0.861–1.114)
Marital Status				
Never Married	1		1	
Married	1.057	(1.015–1.101)	1.030	(0.968–1.096)
Separated	1.215	(1.145–1.290)	1.156	(1.058–1.264)
Divorced or Widowed	1.149	(1.101–1.200)	1.137	(1.063–1.216)
Living Arrangement				
Independent	1		1	
Dependent	1.119	(1.084–1.155)	0.933	(0.883–0.986)
Homeless	1.262	(1.212–1.315)	0.892	(0.823–0.967)
Veteran Status	1.038	(0.955–1.129)	0.910	(0.798–1.037)
Years of Education				
<8 Years	1		1	
9–11 Years	1.076	(1.004–1.152)	1.115	(1.003–1.238)
12 Years	1.051	(0.984–1.122)	1.069	(0.968–1.181)
13–15 Years	1.382	(1.290–1.482)	1.103	(0.991–1.227)
16+ Years				
Employment Status				
Full-Time	1		1	
Part-Time	1.125	(1.065–1.188)	1.209	(1.114–1.313)
Unemployed	1.093	(1.052–1.135)	1.060	(0.970–1.158)
Not in Labor Force	0.864	(0.831–0.899)	1.019	(0.928–1.119)
Primary Source of Income				
Wages/Salary	1		1	
Public Assistance	0.680	(0.639–0.723)	0.956	(0.863–1.059)
Retirement/Pension or Disability	0.691	(0.647–0.737)	0.978	(0.876–1.093)
Other	0.684	(0.645–0.726)	0.966	(0.878–1.062)
None	1.207	(1.163–1.254)	0.894	(0.823–0.970)
Health Insurer				
Private	1		1	
Medicaid	0.522	(0.491–0.555)	0.914	(0.846–0.987)
Medicare or Other	0.871	(0.803–0.945)	0.975	(0.881–1.080)
Uninsured	1.045	(0.982–1.112)	0.964	(0.894–1.039)
Service Setting at Time of Admission				
Hospital	1		1	
Short-Term	0.273	(0.216–0.344)	0.382	(0.284–0.514)
Long-Term	0.389	(0.308–0.491)	0.547	(0.405–0.740)
Ambulatory, Intensive Outpatient	0.180	(0.142–0.227)	0.221	(0.164–0.297)
Ambulatory, Non-Intensive Outpatient	0.133	(0.105–0.167)	0.243	(0.181–0.326)
Primary Source of Referral				
Individual (including Self-Referral)	1		1	
Alcohol/Drug Abuse Care Provider	1.885	(1.802–1.973)	1.322	(1.226–1.426)
Other Health Care Provider	1.536	(1.451–1.626)	1.024	(0.933–1.124)
Educational Institution	1.848	(1.060–3.221)	0.909	(0.317–2.609)
Employer	1.823	(1.332–2.495)	1.414	(0.904–2.210)
Other Community Referral	1.316	(1.260–1.374)	1.789	(1.668–1.918)
Court/Criminal Justice Referral/DUI	2.270	(2.204–2.338)	1.805	(1.716–1.899)
Alcohol or Benzodiazepines Reported at Admission	1.646	(1.599–1.695)	1.226	(1.171–1.283)

*Adjusted for year, state, age, sex, ethnicity, marital status, living arrangement, veteran status, years of education, employment status, primary source of income, health insurer, census division, service setting at time of admission, primary source of referral, and alcohol/benzodiazepine report at admission.

#### Sociodemographic characteristics

Only those aged 21–24 showed higher odds of waiting over a week to enter treatment compared to the reference group of those aged 18–20. No statistically significant difference in the odds of waiting over a week were found between men and women. Black or African Americans showed lower odds of waiting over a week to enter treatment compared to White Americans. Compared to those who were never married, those who were separated, divorced, or widowed showed higher odds of waiting over a week to enter treatment. Those in a dependent living situation or homeless showed lower odds of waiting over a week to enter treatment compared to those who reported living independently. No statistically significant difference was observed in the odds of waiting over a week to enter treatment between veterans and non-veterans. Those working part-time showed higher odds of waiting over a week to enter treatment vis-à-vis those working in full-time employment. Moreover, those reporting no primary source of income showed lower odds of waiting over a week to enter treatment than those reporting a primary income from wages/salary. Those covered by Medicaid showed lower odds of waiting over a week to enter treatment compared to those who were insured privately.

#### Institutional characteristics

Admissions to all examined non-hospital settings were associated with lower odds of waiting over a week to enter treatment. Compared those who were individually referred for treatment (including self-referrals), those who were referred by an alcohol/drug abuse care provider, other community referrer, or the criminal justice system showed higher odds of waiting over a week to enter treatment. In addition, those reporting alcohol or benzodiazepines at admission also showed higher odds of waiting over a week to enter treatment.

## Discussion

Our findings highlight several differences in the receipt of OAT and waiting time to enter treatment on patient sociodemographic, institutional and behavioural characteristics. Firstly, we note that only a minority of patients admitted for OUD receive OAT with some subpopulations exhibiting much lower receipt of OAT than others. For instance, only 18% of those aged 18–20 received OAT compared to almost 60% of patients aged 55 and over who received OAT. Similarly, while approximately three-quarters of admitted patients were treated with no reported wait time, some subpopulations reported differentially higher rates of those waiting for over a week to enter treatment, such as those aged 18–20, those who were privately insured and those admitted to the hospital setting. Some subpopulations showed higher odds of receipt of OAT, including all age groups older than the reference group of patients aged 18–20, Native Americans, patients whose primary source of income was public assistance or retirement/pension or disability funds, those insured on Medicaid or Medicare, and those admitted to non-hospital care settings. By contrast, some groups showed lower odds of receipt of OAT, including those with a marital status other than the reference group who were never married, those in a dependent living situation or homeless, and those patients for whom the primary source of referral was anything other than an individual referral. With respect to covariates associated with increased odds of waiting over a week to enter treatment, our analysis highlighted several groups, including those who were separated, divorced, or widowed, those working part-time, and those who were referred by alcohol/drug abuse care providers, community referrers, or the criminal justice system.

Our analysis is necessarily limited by use of the TEDS-A dataset. Firstly, given the relative complexity of reporting from the facility to the state to the Federal level, variations on reporting mechanisms by state may have downstream effects on the quality of data at the national level [30]. In addition, information on days waiting to enter treatment are collected through TEDS Supplementary Data which is voluntary [19]. As such, facilities with longer waiting times may choose not to submit this information thereby contributing a level of reporting bias to our analysis leading to the underestimation of actual waiting times to enter treatment. Importantly, inferences regarding national trends and patterns are limited given that 7 states did not report any patients in receipt of OAT and 13 states did not report time waiting to enter treatment. Many of these states are critical to accurately assessing these outcomes respectively, at a national level and so our inferences should be interpreted cautiously without their inclusion. Additionally, given that our analyses are limited to only first-time admissions for the treatment of OUD, we do not include subsequent admissions for the treatment of OUD following admissions for detoxification or prior admissions for treatment. We recognise that this may distort our estimates of OAT for the treatment of OUD, given that an individual may be admitted several times before receiving OAT. Moreover, TEDS-A does not include the use of OATs in the primary care setting and, consequently, conclusions regarding the use of OATs in primary care cannot be drawn from our analysis though data on the topic is available in established literature [31–33]. Nevertheless, no other dataset exists at the national level which provides comparable data to TEDS-A. Consequently, despite the limitations presented here, our study draws upon the largest extant dataset to provide information on OAT and time waiting to enter treatment.

Addressing differences in treating individuals affected by OUD is a chief concern for policymakers and care providers. One systematic review of determinants of opioid-related mortality in the United States and Canada has found opioid-related mortality trends tend to vary considerably by sociodemographic differences, including ethnicity, gender, age, and socioeconomic status, as we have highlighted here [34]. For many of these subpopulations, differences in the treatment of OUD occurs concomitantly with differential treatment more generally, exacerbating existing known disparities in healthcare provision based on factors such as race [35]. Indeed, failure to treat OUD must be considered more widely. Perlman and Jordan, for instance, highlight the complex inter-relationships among opioid misuse and overdose, hepatitis C, and HIV as a syndemic with disproportionately adverse results for individuals at heightened risk [36]. These concurrent conditions may further problematize the treatment of OUD and, indeed, may contribute to a myriad of downstream metabolic comorbidities although much remains unknown [37]. In addition, our findings regarding individuals referred by the criminal justice system are consistent with the literature regarding the relatively low uptake of pharmacotherapy for opioid use disorder among incarcerated individuals [38], a subgroup which has exhibited a heightened risk of opioid overdose mortality following post-release [39]. As a result, OUD, taken in context of wider trends in population health, is increasingly an urgent priority and differences in treatment must be addressed both in the near- and long-term.

Our analysis highlights a number of areas for further scrutiny. Firstly, although OAT is widely considered the standard of care for OUD, only a minority of admitted patients receive it. Moreover, variations in who receives OAT and time to enter treatment based on sociodemographic and institutional characteristics highlight further areas for further study and potential intervention. In addition, further research is needed regarding personalised approaches to characterising the inheritable factors which contribute towards heightened risk of OUD as well as potential avenues for more effective treatment [40]. Nevertheless, given the limitations of the TEDS-A dataset, we are unable to unravel the causal mechanisms which underlie these differences. Stigma is commonly cited as a major factor which attenuates greater uptake of OAT for the treatment of OUD but access remains strictly controlled and also contributes to some patterns we have highlighted here [41]. Further attention is warranted to understand how and why these differences exist and persist in order to formulate appropriate policy responses.
